# Perforated intestinal leiomyosarcoma as a metastasis of uterine leiomyosarcoma: a case report

**DOI:** 10.4076/1757-1626-2-9288

**Published:** 2009-09-17

**Authors:** Barış Saylam, Ömer Vefik Özozan, Arife Polat Düzgün, Bahadır Külah, Özge Han, Faruk Coşkun

**Affiliations:** 1 Department of 3rd Surgery, Ankara Numune Teaching and Research Hospital, Ankara, Turkey; 2 Department of Pathology, Ankara Numune Teaching and Research Hospital, Ankara, Turkey

## Abstract

**Introduction:**

Uterine leiomyosarcomas are relatively uncommon soft tissue neoplasms and rarely metastases to small bowel. In the current case; a patient is suffering from intestinal perforation due to metastatic leiomyosarcoma of the small bowel.

**Case presentation:**

A 59-year-old woman underwent a modified radical mastectomy for infiltrating ductal cancer of the breast six years ago and a total abdominal hysterectomy for leiomyosarcoma of the uterus two years ago. About 2400 cGy total dose radiotherapy has also been applied after total hysterectomy for bone metastasis of breast cancer. She admitted to our clinic with the complaints of acute abdomen due to perforated small bowel metastasis of leiomyosarcoma during the radiotherapy. Laparotomy was performed and leiomyosarcoma of the ileum was removed totally. Histopathologic examination of the specimen confirmed the presence of the leiomyosarcoma in intestinal tissue samples.

**Conclusion:**

We aimed to present this unusual case which perforated presentation of the intestinal metastasis of uterine leiomyosarcoma.

## Introduction

Uterine leiomyosarcomas are relatively uncommon soft tissue neoplasms. They mostly metastases to lung and liver tissue, whereas rarely metastases to small bowel, heart, brain and thyroid [1,2].

In this current case the patient's admission complaint was acute abdomen (small bowel perforation) due to leiomyosarcoma of the uterus. Small bowel perforation due to uterine leiomyosarcoma is relatively uncommon. However this is a unique condition, the route of spreading from uterus via small bowel is likely to be related with hematogenous spread.

## Case presentation

A 59-years-old Turkish woman who had undergone modified radical mastectomy for a stage IIB (T2N1M0) infiltrating ductal carcinoma of left breast. After modified radical mastectomy of the left breast, she had a disease free period of four years. Afterwards disease free survival, the patient suffered from menorrhagia. On pelvic examination, an enlarged uterus, the size of which was similar to that at 12 weeks' gestation, was noted and ultrasonographic examination revealed a leiomyoma of the uterus. The patient had underwent a diagnostic laparotomy for a suspicion of leiomyoma. During the evaluation total abdominal hysterectomy, bilateral salpingo-oophorectomy, omentectomy and appendectomy was performed. The uterus was symmetrically enlarged, weighed 650 gr, and measured 13 cm × 12 cm × 6 cm. Uterine tumor measuring 9 × 7 × 5 cm with central necrosis, and there was no obvious disease involving the bilateral ovaries, lymph nodes or omentum The result of pathology was a leiomyosarcoma of the uterus without any metastatic lesion. There was no evidence of uterine serosal and pelvic lymph node involvement. No adjuvant treatment, such as chemotherapy or radiotherapy, was arranged after the surgery.

Sixteen months later the patient had undergone cholecystectomy and incisional hernia repair. During laparotomy there was no evidence of metastasis. Afterwards these operations, during routine controls a metastatic lesion in the lomber vertebraes was found. While the patient was receiving radiotherapy for this metastatic bone lesion. She admitted to the emergency room for abdominal discomfort. Physical examination revealed tenderness and rigidity also labaratory tests were as follows; hemoglobin 9.7 g/dl, white blood count 17 500 mm³, chest and abdominal plain radiography were normal. Abdominal ultrasonography revealed fluid collection in the pelvis.

Then the patient had undergone an emergency surgical operation. A solid necrotic tumoral mass 4 × 5 cm in diameter was found 2 inches proximal to the ileocecal valve. There was also a perforation in the small bowel with generalized peritonitis. The tumoral segment was resected with tumor free margin and also with regional lymph node dissection was performed.

**Figure 1:**
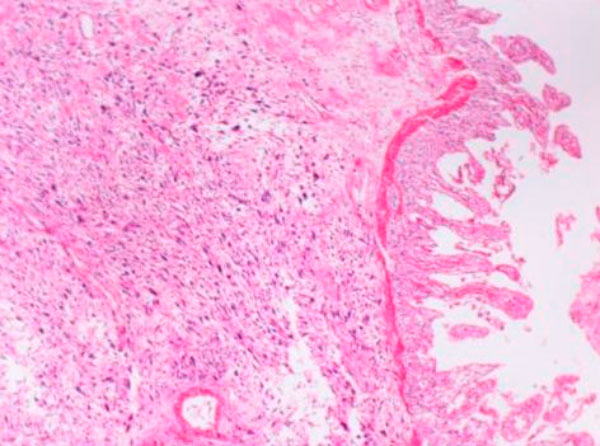
**Histologic appearance of the tumor at the submucosa and muscular layer of the small bowel (HE, x40)**.

**Figure 2:**
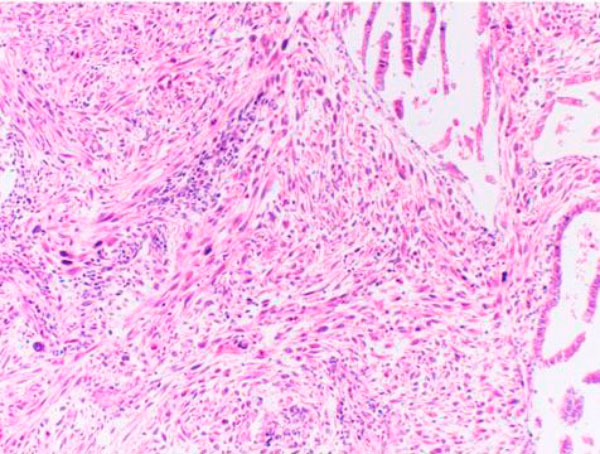
**Histologic appearance of the tumor in the uterus (HE, x200)**.

**Figure 3:**
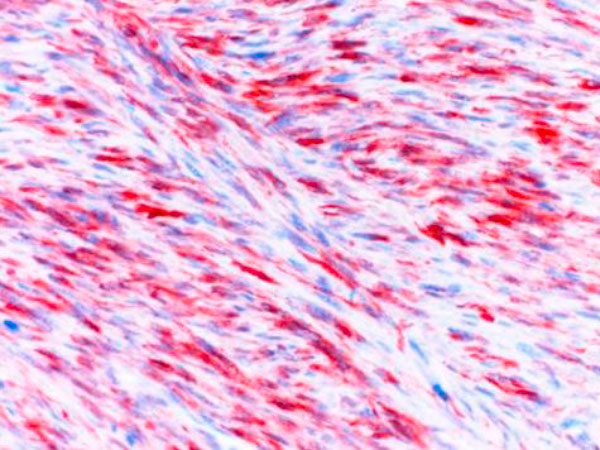
**The tumor cells show a positive reaction for α-smooth muscle actin (x400)**.

Microscopically the tumoral invasion was observed all through the intestine wall from mucosa into the serosa. There was areas of necrosis with spindle cells in an interlacing or fascicular patern (Figure 1). Histopathological findings of the tumoral mass was similar to the one which was resected two years ago (Figure 2). There was a moderate degree of cellular atypia. Mitotic activity ranged from 7-17 per 10 high-power field. The tumor cells showed a strong positive reaction to α-smooth muscle actin (Figure 3) and vimentin. Whereas there was no reaction with keratin, desmin, S-100, myoglobyn, EMA, NSE, CD 31, CD 34, CD 68 and CD117. The diagnosis of leiomyosarcoma was performed according to these reactions. There was no lymph node metastasis the resection margins free of tumor cells.

After 10 months disease free survival the patient admitted to hospital with low gastrointestinal bleeding. During evaluation; a metastatic lesion was evaluated in transverse colon. The patient rejected any kind of medical assistance and died after a short period of time.

## Discussion

Uterine leiomyosarcoma is a rare disease and is difficult to differentiate from benign leiomyoma, even by MRI. Uterine leiomyosarcoma presents symptoms similar to leiomyoma, such as massive uterine bleeding, pollakiuria and abdominal pain, and is frequently asymptomatic. Leibsohn et al. reported that out of 1429 patients who underwent hysterectomies due to presumed benign leiomyoma, leiomyosarcoma was histopathologically diagnosed in seven patients (0.5%) [3].

Leiomyosarcoma of the gastrointestinal tract can either be primary or secondary. Leiomyosarcomas in uteri can invade by direct extension, hematologic route, implantation, or via lymphatic route [2]. Spontaneous perforation of the gastrointestinal tract in patients with metastatic lesion is uncommon but life threatening complication. Torosian and Turnbull reported an operative mortality rate of 53% in cancer patients receiving corticosteroids and chemotheraphy with perforation of the small bowel and colon [4].

Evans et al. reported that all smooth muscle tumors arise from the muscularis propria layer of the intestinal wall [5]. Wilson et al. reviewed 2144 published case of primary malignant tumors of the small bowel; 421 of these were primary leiomyosarcomas. They reported that such tumors nearly always arose from the muscularis propria layer of the intestinal wall [6].

In the current case, the leiomyosarcoma of the intestinal wall was accepted as a metastatic lesion because it arises from the mucosal layer of the intestinal wall; contrary to the primary leiomyosarcomas.

Secondary involvement of the bowel from uterine sarcomas have been reported and accepted as direct implantation by the transperitoneal route [2]. There is only one case report in the literature about metastatic leiomyosarcoma of the uterus with late endobronchial and small bowel metastases by hematogenous route. Patient with metastatic lesion to the small bowel in this case was in the submucosa, and the growth resulted in intramural mass with polypoid extension in to the lumen [7].

Radiotheapy complications involved in the gastrointestinal system are classified into two distinct groups as: early complications, which occur during or immediately after treatment, and late complications, which can occur months or years after radiotherapy. Histopathologic signs of the tissue damage due to the radiotherapy was defined as atrophic alterations in villi and crypts, teleangiectasiae in the submucosa and inflammatory cell infiltrations in submucosal region [8]. These findings are not consistent with the our findings. In histopatologic examination of the perforated intestinal tissue revealed no any signs of damage related with the radiotherapy in this case.

## Conclusion

In conclusion, small bowel recurrence following treatment for uterine leiomyosarcoma without other concomitant metastases is rare, and the route of spread remains uncertain. However, bowel metastasis via lymphatic or haematogeneous route from the primary site in somatic soft tissues is not very rare [9].

Leiomyosarcoma of the uteri may result in a metastatic lesion in the mucosal layer of the intestinal wall and cause of an intestinal perforation as an unusual finding during admission.

## Competing interests

The authors declare that they have no competing interests.

## Consent

Written informed consent was obtained from the patient for publication of this case report and accompanying images. A copy of the written consent is available for review by the Editor-in-Chief of this journal.

## Authors' contributions

BS assisted the surgery, prepared the manuscript. ÖVÖ helped in searching references, assisted drafting the manuscript. BK gave final touch to the manuscript. APD did the surgery. ÖH assisted drafting the manuscript. FC guided during surgery, and in assisted drafting the manuscript. All the authors read and approved the manuscript.
